# Low levels of peripheral blood activated and senescent T cells characterize people with HIV-1-associated neurocognitive disorders

**DOI:** 10.3389/fimmu.2023.1267564

**Published:** 2023-10-25

**Authors:** Lucy Kundura, Renaud Cezar, Manuela Pastore, Christelle Reynes, Jérémy Deverdun, Emmanuelle Le Bars, Albert Sotto, Jacques Reynes, Alain Makinson, Pierre Corbeau

**Affiliations:** ^1^ Institute of Human Genetics, Centre National de la Recherche Scientifique-Montpellier University UMR9002, 141 rue de la Cardonille, Montpellier, France; ^2^ Immunology Department, Nîmes University Hospital, Place du Pr Debré, Nîmes, France; ^3^ Institute of Functional Genomics UMR5203 and BCM, CNRS-INSERM-Montpellier University, 141 rue de la Cardonille, Montpellier, France; ^4^ Institute of Human Functional Imaging, Montpellier University Hospital, Montpellier, France; ^5^ Department of Neuroradiology, Montpellier University Hospital, Montpellier, France; ^6^ Infectious and Tropical Diseases Department, Nîmes University Hospital, Nîmes, France; ^7^ Faculty of Medicine, Montpellier University, Montpellier, France; ^8^ Infectious and Tropical Diseases Department, Montpellier University Hospital, Montpellier, France

**Keywords:** cognition, T cell activation, T cell senescence, inflammation, neuroimaging

## Abstract

**Background:**

HIV infection induces a 75% increase in the risk of developing neurocognitive impairment (NCI), which has been linked to immune activation. We therefore looked for immune activation markers correlating with NCI.

**Method:**

Sixty-five people aged 55-70 years living with controlled HIV-1 infection were enrolled in the study and their neurocognitive ability was assessed according to the Frascati criteria. Fifty-nine markers of T4 cell, T8 cell, NK cell, and monocyte activation, inflammation and endothelial activation were measured in their peripheral blood. White matter hyperintensities (WMH) were identified by magnetic resonance imaging. Double hierarchical clustering was performed for the activation markers and 240 patients including the 65 whose neurocognitive performance had been evaluated.

**Results:**

Thirty-eight percent of volunteers presented NCI. Twenty-four percent of them were asymptomatic and fourteen percent had a mild disorder. Strikingly, activated (HLA-DR+) as well as senescent (CD57+CD28-CD27±) T4 cells and T8 cells were less prevalent in the peripheral blood of participants with NCI than in participants without the disorder. Accordingly, the percentage of HLA-DR+ T4 cells was lower in volunteers with periventricular and deep WMH. The double hierarchical clustering unveiled six different immune activation profiles. The neurocognitive performances of participants with two of these six profiles were poor. Here again, these two profiles were characterized by a low level of T4 and T8 cell activation and senescence.

**Conclusion:**

Our observation of low circulating levels of activated and senescent T cells in HIV-1 patients with NCI raises the interesting hypothesis that these lymphocytes may be recruited into the central nervous system.

## Introduction

Neurocognitive impairment (NCI) may be observed in 20 to 30%, and up to 60% of efficiently treated people living with HIV (PLWH) (1, 2). We recently published an almost 75% increase in the risk of HIV-associated neurocognitive disorder (HAND) in ageing PLWH after adjustment on age, sex, education, comorbidities, depression and social confounding factors (2).

In the course of HIV infection under treatment, NCI may be fuelled by various etiological factors. First, certain antiretroviral drugs may be neurotoxic *per se*, or have important adverse neurological effects (3). Second, depression (4), drug abuse (5) or co-infections by, for instance, cytomegalovirus (6) or hepatitis C virus (7) may also contribute to HAND. However, major potential drivers of NCI are metabolic disorders and atherothrombosis. Atherothrombosis is common in PLWH (8) and linked to neurocognitive disorders, both in the general population (9) and in PLWH (10–12). In line with the role of atherosclerosis in HIV-induced NCI, an earlier decrease in cerebral blood flow has been shown in PLWH than in healthy people (13). White matter hyperintensity (WMH) identified by magnetic resonance imaging (MRI) may also be associated with cerebrovascular disorders (14). An increase in WMH has been reported in PLWH, and linked to NCI, even in the setting of well-controlled infection (15, 16). After controlling for age and history of tobacco use, Mina et al. observed that PLWH had an almost 4-fold higher chance of increased WMH compared to controls without HIV (16). Seider et al. showed the importance of age in HIV-associated white matter damage (17). Another major potential cause of NCI is neurodegeneration (18), more frequently observed in PLWH than in non-infected individuals (19). Here, HIV persistence in the central nervous system (CNS) (20), particularly in microglial cells (21) seems to play a role (22). HIV has direct toxic effects on the CNS via its components, including trans-activators of transcription (tat), viral protein R and glycoprotein120 (23). It also has indirect toxic effects via the production of inflammatory cytokines and chemokines, excitotoxins and nitric oxide radicals it induces (24). Thus, microglial activation has been shown to be inversely linked to cognition (25). Globally, NCI has been linked to systemic monocyte activation and inflammation in aviremic PLWH under treatment. For instance, high percentages of CD38-positive (26) and IL-1β-expressing monocytes (27), as well as high levels of circulating soluble CD14 (28) and soluble CD163 (28, 29) have been reported in cognitively impaired patients. Likewise, peripheral blood concentrations of the inflammatory chemokine MCP-1 has been linked to HAND (30).

It is interesting to note that immune activation may drive other causes of NCI, since it is known to favour metabolic disorders (31), depression (32), atherothrombosis and neurodegeneration (33), and also limit neurogenesis (34).

With the aim of better defining the types of immune activation linked to NCI in PLWH, we used supervised and non-supervised global approaches to look for biomarkers associated with clinical and visual signs (MRI) of NCI among 80 peripheral immune activation markers. To our surprise, NCI was preferentially characterized by low levels of circulating activated and senescent T cells. These findings raise the interesting hypothesis that T cell recruitment in the CNS might play a pivotal role in NCI.

## Materials and methods

### Study design

This is a substudy of the ANRS EP58 HAND 55-70 study which evaluated the prevalence of NCI in PLWH on efficient antiretroviral therapy (2). Patients were sequentially recruited at the University Hospitals of Montpellier and Nîmes. Inclusion criteria were the following: age 55-70 years, controlled HIV-1 infection (<50 copies/mL and less than 2 viremic blips) for at least 24 months, and CD4 count ≥200 cells/μL. Confused, illiterate, vulnerable, or individuals who were non-fluent in French, and individuals with brain, sensorial, or psychiatric disease were not included. The CPP Sud Méditerranée I Ethics Committee (Marseille, France) had approved this study. All patients had provided written informed consent.

### Cognitive and functional evaluations

Neuropsychologists carried out the following tests: the trail-making test (TMT) A-B, the digital symbol substitution task of the Wechsler Adult Intelligence Scale-IV (WAIS-IV), the digital finger-tapping test, word fluency and formal lexical and semantic evocation and the free and cued selective reminding test as previously described (2). Functional capacities were evaluated with the instrumental activities of daily living (IADL) scale. Presence of NCI was assessed according to the Frascati criteria (35). In asymptomatic neurocognitive impairment (ANI) and mild neurocognitive disorder (MND), there is mild neuropsychological impairment in at least 2 domains of ability. ANI has no negative effect on everyday life, whereas MND results in 2 or more signs of decreased everyday functioning.

### Flow cytometry

Monoclonal antibodies conjugated with fluorescein isothiocyanate (FITC), phycoerythrin (PE), energy-coupled dye (ECD), PE-Cyanine5.5 (PC5.5), PE-Cyanine7 (PC7), Alexa Fluor 647 (AF647), allophycocyanine (APC), APC/Alexa700, or APC/Alexa750 were purchased from Beckman Coulter. The antibodies were used in the following combinations; CD57-FITC/CD279-PE/CD45RA-ECD/C28-PC5.5/CD27-PC7/CD8-APC/CD4-APC700/CD3-APC750, CD8-APC/CD4-APC700/CD3-APC750/CD38-PE/HLADR-PC7, CD3-APC750/CD16-APC/HLA-DR-PC7/CD56-PC5.5/CD14-PE/CD57-FITC, CD4-FITC/CD45RA-ECD/CD25- PC7/FOXP3-AF647/CD127-APC750. Whole blood collected in EDTA tubes was stained within one hour for 10 minutes at room temperature in the dark with a cocktail of antibodies and fixed using an IMMUNOPREP reagent system kit and TQ Prep automate (Beckman Coulter) according to the manufacturer’s protocol. For FoxP3 intracellular labelling, cells were permeabilized and fixed with PerFix-nc kit (Beckman Coulter) according to the manufacturer’s guidelines. Cells were run on a Navios flow cytometer and results were analyzed using Kaluza software (Beckman Coulter). A minimum of 20,000 lymphocytes were gated to analyze the subpopulations. We controlled the inter-run variability with the same batch of FlowSet Pro Beads (Beckman Coulter). During the study, no voltage adjustment was necessary to keep the beads in their respective defined targets. We have previously described the gating strategy (36), which is exemplified in the **Supplementary Figure 1**.

### Soluble markers in peripheral blood

ELISA was used to quantify soluble TNF receptor I (sTNFRI), soluble CD163 (sCD163) (Quantikine, R&D systems), as well as tissue Plasminogen Activator (tPA), and soluble Endothelial Protein C Receptor (sEPCR) (Asserachrom, Stago, USA) in plasma collected in EDTA vacutainer tubes (Becton Dickinson) and frozen.

### Magnetic resonance imaging

Neuroimaging data were collected on a 3T MRI scanner (MAGNETOM Skyra, Siemens Healthcare, Germany) using a 32-channel head coil. A T1 anatomical image was acquired using a sagittal 3D magnetization-prepared acquisition with a gradient-echo (MPRAGE, TE = 2.9 ms, TR = 2300 ms, TI = 900 ms, flip angle = 9°, voxel size = 0.98 x 0.98 x 1.2 mm^3^, number of slices = 176). To evaluate WMH, an additional sagittal 3D T2-FLAIR acquisition was performed (TE = 400 ms, TR = 5000 ms, TI = 1800 ms, turbo factor = 270, voxel size = 0,5 x 0.5 x 1,1 mm^3^, number of slices = 144).

WMH were rated using the Fazekas visual scale. The Fazekas score describes the different types of hyperintense signal abnormalities observed. A score of 0 to 3 was used for periventricular hyperintensities (0 = no lesions, 1 = pencil thin lining, 2 = smooth halo, 3 = irregular with extension into deep white matter) and 0 to 3 for deep WMH (0 = no lesions, 1 = punctate foci, 2 = beginning confluence of foci, 3 = large confluent areas) (37). All 3D T2-FLAIR images were reviewed and scored by two neuroradiological observers, and combined in the event of discordance.

### Statistical analysis

Version 4.1.2 of R software was used to perform the statistical analysis. Markers with missing values of more than 10% were discarded. We imputed the remaining missing values with the mean value of the two closest neighbors according to Euclidean distance. Subsequently, when markers presented a correlation higher than 95%, only the marker that best differentiated between controls and neurocognitive troubles, according to a Student t-test, was retained. Since not all markers satisfied normality, we ran a non-parametric Kruskal Wallis test to select only those that were significantly differently expressed in patients with neurocognitive disorders versus controls. Multiple test correction was performed to control the False Discovery Rate (FDR) using the Benjamini-Hochberg method. Linear Discriminant Analysis (LDA) was applied to each immunological marker combination using the MASS R package (36). To avoid over-fitting, cross-validation was used to evaluate the accuracy of prediction. To select an optimal number of variables and create a parsimonious predictive model of markers selected with the previous filter, we chose genetic algorithms (36, 38). The genetic algorithm was run four times and all solutions of the final generations were evaluated through 30 runs of independent linear discriminant analysis with 2-fold cross validation. Solutions were ranked according to their average correct classification rate during the cross-validation process. As previously described (36), clustering analysis was carried out for patients, using the Euclidian distance to measure the distance between individuals, and another for markers, using 1-abs (correlation) as a distance. For both of them, Ward’s minimum variance method was used as a linkage method. We then generated a heat map based on patient classification and markers. The possibility to cluster the data was assessed using principal component analysis, and also by seeking a cluster structure in the distance matrix. The Hopkins statistic was calculated, with a value of 1 indicating the highest possibility to cluster the data. We determined the optimal number of clusters using Silhouette and Gap statistic.

## Results

### Study subjects

Sixty-five adults living with HIV-1 for a mean (SD) duration of 20.0 (8.0) years, were recruited at the University Hospitals of Nîmes and Montpellier, France. Eleven percent of them were females, and 89% males. Their mean age was 62.2 (4.0) years. They were of European, African, and Asian origin for 88%, 9% and 3%, respectively. Their pretherapeutic CD4 count was 168 (140) cells/μL, their current CD4 count was 633 (245) cells/μL, and their current CD4/CD8 ratio 0.92 (0.46). All had undetectable viral loads, except for a maximum of two blips (transient elevation of viral load ≥ 200 copies/mL). Their educational level was grade school (15%), high school (49%), or college (36%) and thirty-one of them were being treated for depression. Frequencies of tobacco and drug consumption were 42% and 38%, respectively. Eight percent were HBV-infected and fifteen percent HCV-infected (**Table 1**). Their prevalence of diabetes, high blood pressure and cardiovascular disease is indicated in **Table 1**.

**Table 1:** Bioclinical characteristics of the volunteers.

Characteristic	All volunteers (N=65)	No NCI (N=40)	ANI (N=16)	MND (N=9)	p
Age (years) Mean (SD)	62.2 (4.0)	62.5 (4.1)	61.5 (4.0)	62.3 (4.4)	0.699
Sex					
Female (%)	11	7	12	22	
Male (%)	89	93	88	78	0.422
Ethnicity					
Asian (%)	3	5	0	0	
African (%)	9	5	19	11	
Caucasian (%)	88	90	81	89	0.443
Education level					
Primary (%)	15	12	12	33	
Secondary (%)	49	53	50	34	
Superior (%)	36	35	38	33	0.597
Physical activity					
Null (%)	6	5	0	25	
Faint (%)	27	22	20	63	
Medium (%)	38	38	60	0	
Intense (%)	29	35	20	12	0.112
Tobacco consumption (%)	42	33	70	33	0.120
Alcohol consumption (%)	23	15	35	37	0.147
Drug consumption (%)	38	42	37	22	0.526
Depression (%)	18	12	25	33	0.256
Diabetes (%)	15	15	12	25	0.689
High blood pressure (%)	34	40	29	12	0.293
Cardiovascular disease (%)	25	20	29	37	0.500
HBV infection (%)	8	12	0	0	0.184
HCV infection (%)	15	12	12	33	0.275
Pre-therapeutic CD4 count (cells/μL) Mean (SD)	168 (140)	147 (120)	170 (111)	258 (228)	0.225
Infection duration (years) Mean (SD)	20.0 (8.0)	21.0 (7.7)	18.6 (8.3)	18.3 (9.2)	0.478
Current CD4 count (cells/μL) Mean (SD)	634 (245)	620 (216)	656 (317)	652 (238)	0.860
Current CD4:CD8 ratio Mean (SD)	0.92 (0.46)	0.91 (0.46)	0.95 (0.55)	0.93 (0.33)	0.803
ART regimen					
NRT1^1^ (%)	86	87	94	62	0.104
NNRT1^2^ (%)	33	32	37	25	0.826
P1^3^ (%)	30	35	19	25	0.463
INSTI^4^ (%)	53	52	50	62	0.839

^1^P-value between the 3 patient groups (no NCI, ANI, and MND) calculated using Kruskal Wallis test. ^1^NRTI, nucleotide reverse transcriptase inhibitor; ^2^NNRTI, non-nucleotide reverse transcriptase inhibitor; ^3^PI, protease inhibitor; ^4^INSTI, integrase strand transfer inhibitor.

### Correlations between activation markers and clinical NCI

Thirty-eight percent of participants were classified as HAND, 24% with ANI and 14% with MND; none presented dementia. There was no difference in CD4 nadir between volunteers with ANI or MND or without NCI (p = 0.390). Presence or absence of ANI or MND was neither linked to antiretroviral therapy including a nucleotide reverse transcriptase inhibitor (p = 0.104), a non-nucleotide reverse transcriptase inhibitor (p = 0.826), a protease inhibitor (p = 0.463), nor an integrase strand transfer inhibitor (p = 0.839).

We determined the proportions and absolute numbers of the following subpopulations: (i) activated (CD38+, CD38hi, and/or HLA-DR+), exhausted (PD-1+), senescent (CD57+, eventually CD27- and eventually CD28-), naïve (CD45RA+CD27+), central (CD45RA-CD27+) and effector (CD45RA-CD27-) memory CD4+ and CD8+ T cells, and (ii) activated (HLA-DR+), dysfunctional (CD56-), and senescent (CD57+) NK cells. Monocyte activation was evaluated by measuring sCD163. Inflammation was monitored by quantifying sTNFRI. tPA, and sEPCR were used as markers of endothelium activation. Fifty-nine activation markers were thus quantified.

**Figure 1:**
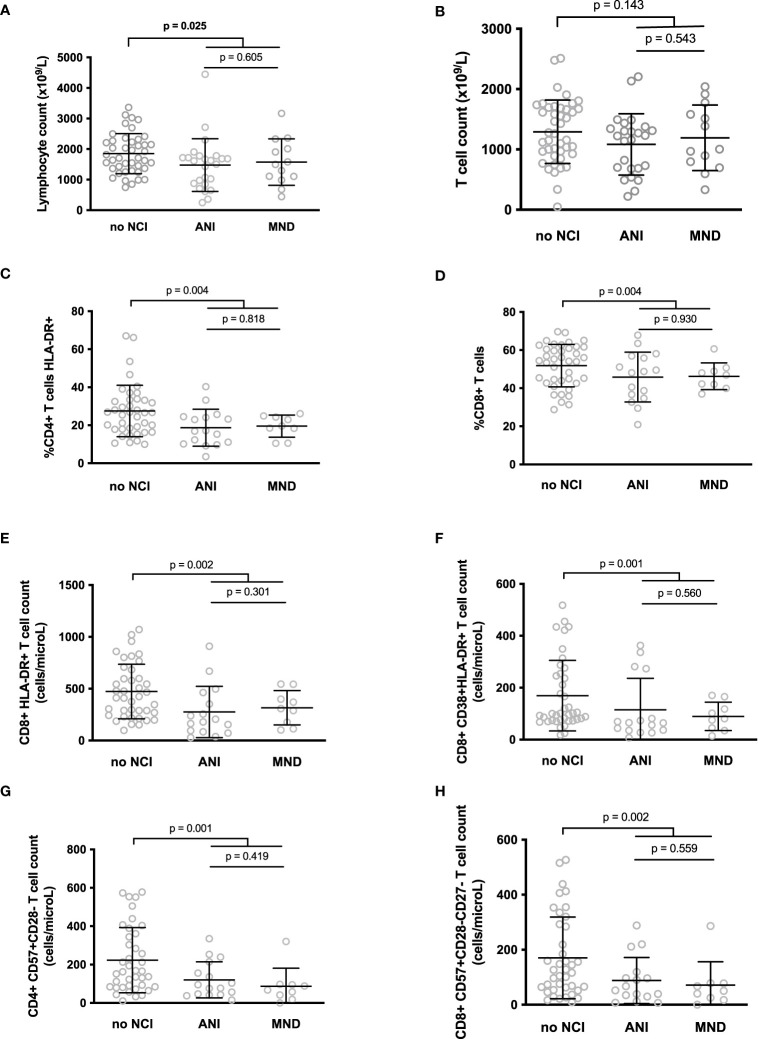
Differences between the lymphocyte **(A)** and T cell **(B)** counts, and between the level of various T cell markers of activation or senescence in participants with neurocognitive impairment (NCI+) or without neurocognitive impairment (NCI-) **(C–H)**. P-values were calculated using a two-sided unpaired student’s *t* test or Mann-Whitney test as appropriate.

We searched for differences in the various markers we measured between volunteers with or without cognitive impairments. Participants without NCI tended to present higher lymphocyte (1851 ± 656 vs. 1511 ± 820 cells/μL, p = 0.025, adjusted p non-significant, **Figure 1A**) and T cell + (1292 ± 526 vs. 1120 ± 516 cells/μL, p = 0.143, adjusted p non-significant, **Figure 1B**) counts than participants with ANI or MND. Strikingly, the proportion of activated, HLA-DR+, CD4+ T cells (19.0 ± 8.4 vs 27.5 ± 13.5%, p = 0.004, adjusted p = 0.049, **Figure 1C**), was lower in patients with ANI or MND than in patients without any NCI. Concerning T8 cells, the percentage of CD8+ T cell (46.0 ± 11.1 vs. 51.8 ± 11.1%, p = 0.004, adjusted p = 0.049, **Figure 1D**), and the number of activated, HLA-DR+ (290 ± 219 vs. 473 ± 263 cells/μL, p = 0.002, adjusted p = 0.043, **Figure 1E**), and HLA-DR+CD38+ (106 ± 102 vs 169 ± 136 cells/mL, p = 0.001, adjusted p = 0.039, **Figure 1F**) CD8+ T cells was also lower in individuals with NCI than in individuals without. In addition, senescent, CD57+CD28- CD4+ (108 ± 93 vs 223 ± 170 cells/μL, p = 0.001, adjusted p = 0.039, **Figure 1G**) and CD57+CD28-CD27- CD8+ (82 ± 83 vs 170 ± 148 cells/μL, p = 0.002, adjusted p = 0.043, **Figure 1H**) were less common in the peripheral blood of NCI patients than in patients without neurocognitive disorders.

In the search for potential confounding factors, we used linear regression to test whether age, sex, education level, depression or alcohol consumption were associated with NCI in our small cohort. None of these variables were linked to ANI or MND (data not shown).

A linear discriminant analysis showed that these 6 biomarkers (percentages of CD8+ T cells and HLA-DR+ CD4+ T cells, numbers of HLA-DR+ CD8+ T cells, HLA-DR+CD38+ CD8+ T cells, CD57+CD28- CD4+ T cells and CD57+CD28-CD27- CD8+ T cells) were able to predict the presence of NCI with an accuracy of 77%. Moreover, a genetic algorithm analysis revealed that only two markers, the percentage of HLA-DR+ CD4+ T cells and the number of CD57+CD28- CD4+ T cells were able to predict ANI and MND with 73% accuracy, 76% sensitivity, and 70% specificity. Other solutions are indicated in **Table 2**.

**Table 2:** Results of genetic algorithm.

Solutions	Marker 1	Marker 2	Marker 3	Number of markers	Prediction accuracy	Sensitivity	Specificity
2	CD57+CD28-CD27- T8 cell count	%T4 HLA-DR+CD38+		2	0,723	0,759	0,757
3	CD57+CD28-CD27- T8 cell count	%T4 HLA-DR+		2	0,721	0,759	0,730
4	CD57+CD28-CD27- T8 cell count	%T4 HLA-DR+	HLA-DR+CD38- T8 cell count	3	0,707	0,759	0,757
5	CD57+CD28-CD27- T8 cell count	%T4 HLA-DR+CD38+	%T4 HLA-DR+	3	0,708	0,793	0,757
6	CD57+CD28-CD27- T8 cell count	%T4 HLA-DR+	%T4 HLA-DR+CD38-	3	0,707	0,793	0,757
7	CD57+CD28- T4 cell count	%T8 HLA-DR+CD38+	%T4 HLA-DR+CD38+	3	0,707	0,759	0,757
8	CD57+CD28-CD27- T8 cell count	%T4 HLA-DR+	CD57+CD28- T4 cell count	3	0,695	0,759	0,703

### Correlations between activation and WMH

**Figure 2:**
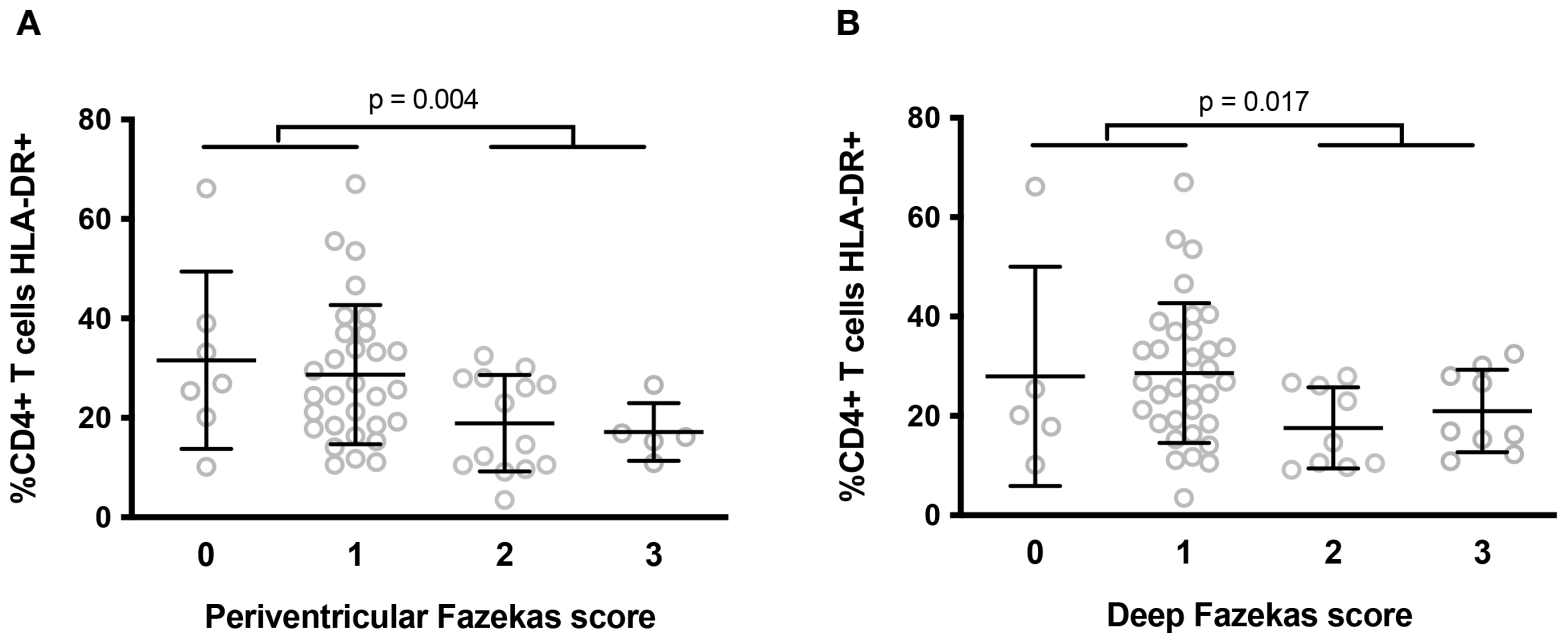
Differences in percentages of peripheral blood activated (HLA-DR+) T4 cells according to the intensity of periventricular **(A)** and deep **(B)** white matter lesions in the central nervous system. P-values were calculated using a two-sided unpaired student’s *t* test or Mann-Whitney test as appropriate.

We also used magnetic resonance imaging to analyze periventricular and deep WMH in the central nervous system based on 3D FLAIR images. Lesions were scored using the Fazekas scale in 56 participants. For periventricular lesions, 12, 54, 25, and 9% of participants scored 0, 1, 2, and 3, respectively. For deep lesions, 9, 59, 16, and 16% participants scored 0, 1, 2, and 3, respectively. Periventricular and deep white matter lesions were strongly correlated (r = 0.891, p < 10^-4^). We then sought correlations between the 6 activation markers identified as being linked to NCI and WMH. We observed that the percentage of HLA-DR+ T4 cells was higher in volunteers with a periventricular (29.2 ± 14.6 vs 18.5 ± 8.7%, p = 0.004, **Figure 2A**) or a deep (28.6 ± 15.0 vs. 19.3 ± 8.2%, p = 0.017, **Figure 2B**) Fazekas score of 0 or 1 than in volunteers a with a periventricular Fazekas score of 2 or 3.

### Identification of immune activation profiles presented by 240 immunological responders

In a group of 140 virological responders, we previously showed that different immune activation profiles may be distinguished using a double hierarchical clustering analysis (39). Such a global unsupervised approach offers an opportunity to have a look at the links between causes, phenotypes, and consequences of immune activation. Indeed, we have observed that some of these profiles may be linked to different sources of immune activation, as microbial translocation (39) or residual viremia (40). Moreover, some of these profiles could pave the way to some comorbidities, as insulin resistance for instance (41). Therefore, we wanted to test whether NCI might be linked to one immune activation profile. To this aim, we added the activation marker values of 100 PLWH, in order to increase the robustness of the immune activation profiles, including the 65 volunteers for whom we had measured neurocognitive ability to those of the 140 PLWH we had previously analyzed to reach a total of 240 PLWH for our analysis. These 41 females and 199 males were 56.4 (9.2) years old. They had been living with HIV-1 for 16.5 (8.5) years. Their pre-therapeutic CD4 counts and current CD4 counts were 185 (138) and 710 (355) cells/mL, respectively. They presented a CD4/CD8 ratio of 1.07 (0.76).

**Figure 3:**
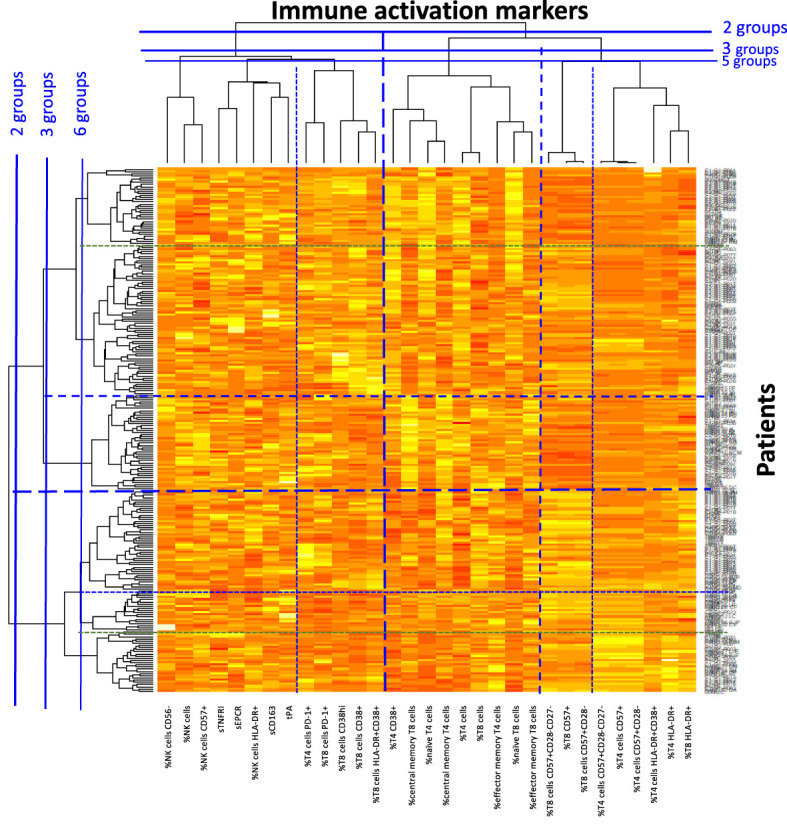
Heat map showing the hierarchical clustering of activation markers as well as virological responders according to their activation profile.

As previously described (36, 39–42), we then performed a double hierarchical clustering analysis again, using the following activation markers: sCD163, sTNFRI, tPA, sEPCR, the percentage of activated (CD38+, CD38hi, and/or HLA-DR+), exhausted (PD-1+), senescent (CD57+, eventually CD27- and eventually CD28-), naïve (CD45RA+CD27+), central (CD45RA-CD27+) and effector (CD45RA-CD27-) memory CD4+ and CD8+ T cells, as well as activated (HLA-DR+), dysfunctional (CD56-), and senescent (CD57+) NK cells. Percentages were preferred to absolute numbers as these are more stable over time. **Figure 3** shows that 6 different immune activation profiles could be identified in these 240 patients. In this heat map, activation markers were classified vertically and patients horizontally. Activation markers which tend to be increased or decreased simultaneously were classified close to each other, whereas independent markers were separated from one another. Patients in the same horizontal cluster (“Profile”) are characterized by the same marks of immune activation.

**Figure 4:**
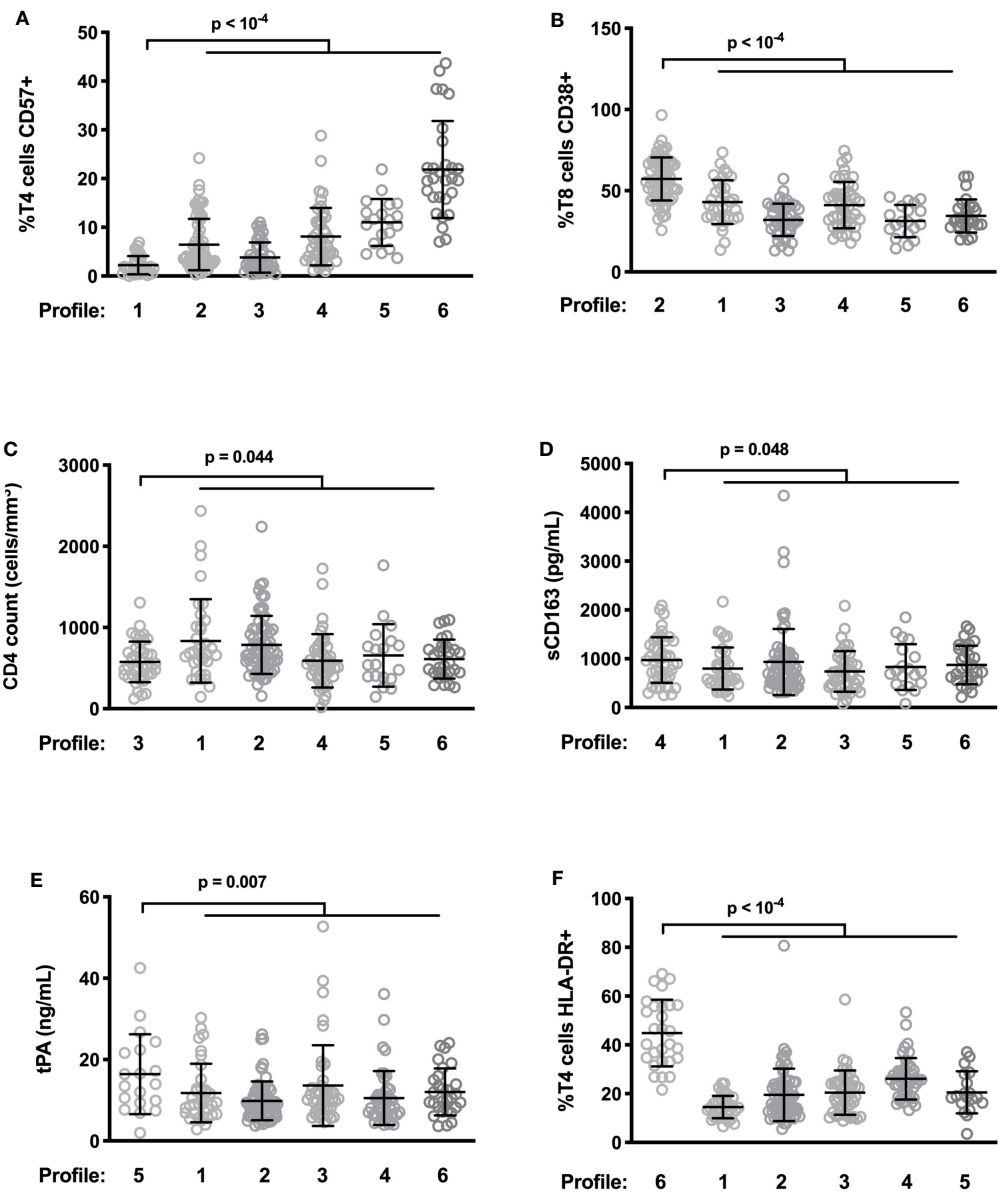
Differences in levels of characteristic activation markers between patient profiles. Frequency of CD57-expressing T4 cells **(A)** and of CD38-expressing T8 cells **(B)**, CD4 count **(C)**, plasma level of sCD163 **(D)** as well as tPA **(E)**, and percentage of HLA-DR+ T4 cells **(F)** in the different participant groups are shown.

The hierarchical clustering gathered patients according to their type of immune activation. We looked for one specific marker able to characterize each profile. Patients with Profiles 1 and 2 had the lowest percentages of CD4+ T cells expressing the senescent marker CD57 (2.2 ± 1.9 versus 8.8 ± 8.1%, p < 10^-4^, **Figure 4A**), and the highest percentages of CD8+ T cells expressing the activation marker CD38 (57.3 ± 13.3 versus 37.1 ± 12.8%, p < 10^-4^, **Figure 4B**), respectively. In Profile 3 patients, it was the low CD4 count that was most remarkable (576 ± 250 versus 712 ± 382%, p = 0.044, **Figure 4C**). Patients with Profiles 4 and 5 had the highest levels of the monocyte activation marker sCD163 (974 ± 466 vs. 858 ± 535 pg/mL, p = 0.048, **Figure 4D**) and the highest levels of the endothelial activation marker tPA (16.4 ± 9.8 versus 11.3 ± 7.0%, p = 0.007, **Figure 4E**), respectively. Finally, Profile 6 was characterized by the highest proportions of CD4+ T cells expressing the activation marker HLA-DR (44.9 ± 13.7 versus 20.4 ± 9.6%, p < 10^-4^, **Figure 4F**).

### Characterization of immune activation profiles linked to neurocognitive disorder

**Figure 5:**
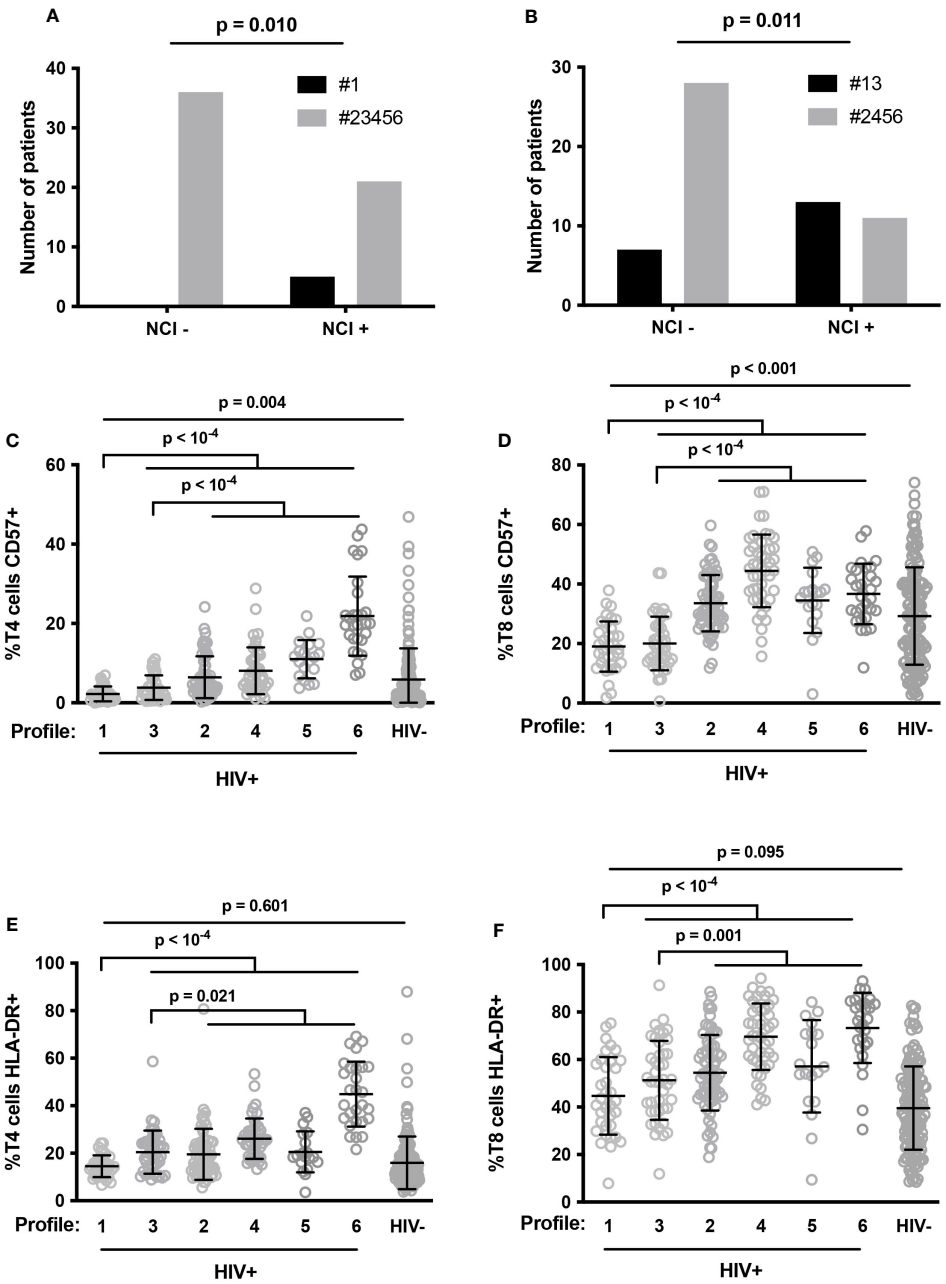
Number of patients with neurocognitive impairment (NCI+) or without it (NCI-) in Profile 1 (#1, closed histogram) and other Profiles (#23456, shaded histogram) **(A)**, and in Profile 1 and 3 (#13, closed histogram) and other Profiles (#2456, shaded histogram) **(B)**. P-values were calculated using Fisher’s test. Proportions of senescent T4 **(C)** and T8 **(D)** cells, and activated T4 **(E)** and T8 **(F)** cells in HIV volunteers (HIV+) with different immune activation profiles and in healthy controls (HIV-). P-values were calculated using two-sided unpaired student’s *t* test or Mann-Whitney test as appropriate.

Next, we focused on the 65 PLWH whose neurocognition had been evaluated. Compared with the other volunteers, patients with Profile 1 more often presented neurocognitive disorders (odds ratio 18.67, 95% CI [0.984; 354.5] (p=0.010), **Figure 5A**). As Profile 1 is particularly associated with neurocognitive impairment, we further characterized this Profile by searching for additional immune activation markers specific to it. In addition to a low proportion of senescent T4 cells (**Figure 5C**), Profile 1 volunteers were remarkable for their low frequency of senescent T8 cells (19.6 ± 8.7 versus 37.3 ± 11.4%, p < 10^-4^, **Figure 5D**), as well as the low frequency of activated (HLA-DR+) T4 cells (14.5 ± 4.6 versus 24.8 ± 13.2%, p < 10^-4^, **Figure 5E**) and T8 cells (44.7 ± 16.3 versus 60.0 ± 17.9%, p < 10^-4^, **Figure 5F**). We compared these percentages with the standard values we had previously established in an age-matched general population (36). Strikingly, Profile 1 patients had proportions of activated T4 cells (**Figure 5E**) and T8 cells (**Figure 5F**) similar to those of controls and proportions of senescent T4 cells (2.2 ± 1.9 versus 5.9 ± 7.8%, p = 0.004, **Figure 5C**) and T8 cells (19.0 ± 8.4 versus 29.2 ± 16.4%, p < 0.001, **Figure 5D**) that were even lower than those of controls. Another group of patients, with Profile 3, had the second highest frequency of NCI (53%). Thus, Profile 1 and 3 patients more often presented neurocognitive disorders than Profile 2, 4, 5 or 6 patients (odds ratio 4.727, 95% CI [1.492; 14.98] (p=0.010), p = 0.011, **Figure 5B**). Here again, compared with Profile 2, 4, 5, and 6 patients, patients with Profile 3 were characterized by a low percentage of HLA-DR+ T4 cells (20.5 ± 9.1 versus 25.9 ± 13.9%, p = 0.021, **Figure 5E**), HLA-DR+ T8 cells (51.2 ± 16.6 versus 62.3 ± 17.5%, p = 0.001, **Figure 5F**), CD57+ T4 cells (3.8 ± 3.1 versus 10.2 ± 8.5%, p < 10^-4^, **Figure 5C**), and CD57+ T8 cells (20.0 ± 9.0 versus 37.3 ± 11.4%, p < 10^-4^, **Figure 5D**).

## Discussion

In this study using independent supervised and non-supervised clustering, we unveiled the low frequency of activated and senescent T4 and T8 cells in the peripheral blood of HIV patients with NCI. In addition, we observed that WMH were more common in PLWH with low frequencies of activated T4 cells. The main explanation for this paradoxical observation is that these T cell subpopulations are rare in the blood because they have been recruited into the central nervous system. Immune cells do patrol the central nervous system (43). Moreover, activated lymphocytes are known to cross the blood brain barrier more efficiently than non-activated lymphocytes (44). In consonance with this, the presence of lymphocytes in the brain and cerebrospinal fluid (CSF) is increased in HIV infection (45), particularly in people with NCI (46). This phenomenon may be exacerbated in immune reconstitution inflammatory syndrome (47). Shacklett et al. reported that T8 lymphocytes in the CSF expressed higher cell surface levels of very late antigen (VLA)-4, leukocyte function antigen (LFA)-1, CCR5 and CXCR3 than circulating T8 lymphocytes in PLWH (48). Accordingly, they also observed a high concentration of the CXCR3-binding chemokine CXCL10 in CSF (48). Price et al. proposed a push and pull model (49) to account for this observation. The “pull” component would be due to chemokines, including CCL3, CCL4, CCL5, and CXCL10, produced in the brain as a consequence of the presence of the virus, attracting CCR5- and CXCR3-expressing T cells. The “push” component would be the consequence of HIV-driven immune activation increasing blood brain barrier permeability and T cell surface expression of adhesion molecules as VLA-4 (α4β1) and LFA-1 (CD11a/CD18) facilitating the crossing of this barrier. It should be noted that CD11a is overexpressed in PLWH (50).

As T lymphocytes in the CSF present activation markers on their surfaces (51, 52), it is logical to assume that the peripheral depletion in activated and senescent T cells we noticed in neurocognitively-impaired patients is due to their transfer to the central nervous system.

Interestingly, lymphopenia has been reported in other NCI diseases like Alzheimer’s disease for instance (53–56). In this neurodegenerative disease, circulating lymphocytes infiltrate into the CNS, where their number increases (57), and the peripheral blood lymphocyte count has been positively associated with cognition and negatively with brain damage (58). Likewise, a low lymphocyte count is predictive of the onset of Parkinson’s disease (59), and T lymphopenia correlates with Parkinson’s disease severity (60). Baseline T lymphopenia even correlated with subsequent cognitive decline in Parkinson disease, but only in patients carrying the ApoE ε4 allele, an allele responsible for permeability of the blood brain barrier (61). This last observation adds credit to the idea that T cell entry into the CNS drives peripheral T cytopenia. T lymphopenia has also been reported in vascular dementia and frontotemporal dementia (62). It may be noted that, in these diseases, lymphopenia is linked to blood brain barrier damage (62), thus further supporting our hypothesis of a causal link between CNS recruitment and peripheral T cytopenia.

Activated and senescent T cells overexpress CCR5, CXCR3 (63, 64), LFA-1, and VLA-4 (65–68). So, given the role of these adhesion molecules and chemokine receptors in the recruitment of T cells in the CNS, it is not surprising that activated and/or senescent T cells are preferentially found in the brain and, therefore, depleted in the circulation.

It would also be of interest to look for the drivers of activated and senescent T cell entry into the CNS. For the “pull” component, any increase in the CNS concentration of CCR5 and/or CXCR3 ligands might preferentially attract activated and senescent T cells. As tat (69) and nef viral proteins induce CXCL10 production in the CNS (70), the presence of HIV in the brain might be a driver. It is also possible that the recruitment of peripheral blood mononuclear cells into the CNS via CXCR3, known to be triggered by amyloid-β accumulation in the brain (71), is exacerbated in HIV patients. For the “push” component, LFA-1 and VLA-4 inducers should be sought. This hypothesis could be tested in a SHIV-infected non-human primate model by infusing T cells overexpressing CCR5, CXCR3, LFA-1, and/or VLA-4 T, and monitoring intracerebral entry and cognitive performances. In humans, a negative correlation between the frequency of CCR5-, CXCR3-, LFA-1-, and/or VLA-4-1-expressing T cells in blood and cerebrospinal fluid could be looked for.

Alternative explanations for the low level of circulating activated and senescent T cells in NCI patients we show here appear to be less likely. Lee et al. reported that people recently infected with HIV-1 present low levels of CD57+CD28- T8 cells, and that this low level was predictive of mortality (72). They hypothesized that this was due to a defect in T8 proliferation and differentiation. Yet, they observed that antiretroviral therapy restored this T8 subpopulation, and the PLWH that we analyzed in our study had been on treatment for an extended period of time. Another hypothesis would be, for any cause including genetics, that the emergence of activated and senescent T cells and/or their life expectancy is lower in HAND patients than in NCI-free patients. But this scenario ought to prevent rather than favor brain damage.

Our work has several limitations. In particular, we analyzed the neurocognition of a limited number of PLWH and, as stated in the introduction, the pathophysiology of NCI is probably multiple. It should also be underlined that our study was transversal, unveiling correlations, but not causality.

However, our data are compatible with the interesting hypothesis that activated and senescent T cells migrating into the CNS might fuel NCI. Accordingly, it would be of interest to test whether a low activated and senescent T cell blood count might be predictive of the onset and progression of NCI, in the same way as T lymphopenia in Parkinson’s disease.

## Data availability statement

The original contributions presented in the study are included in the article/**Supplementary Material**. Further inquiries can be directed to the corresponding author.

## Ethics statement

The studies involving humans were approved by Ethic comity CPP Sud Méditerranée. The studies were conducted in accordance with the local legislation and institutional requirements. The participants provided their written informed consent to participate in this study.

## Author contributions

PC: Conceptualization, Methodology, Supervision, Writing – original draft. LK: Investigation, Validation, Writing – review & editing. CR: Investigation, Validation, Writing – review & editing. MP: Investigation, Methodology, Writing – review & editing. CR: Conceptualization, Supervision, Validation, Writing – review & editing. JD: Investigation, Validation, Writing – review & editing. EL: Conceptualization, Supervision, Validation, Writing – review & editing. AS: Supervision, Validation, Writing – review & editing. JR: Supervision, Validation, Writing – review & editing. AM: Conceptualization, Supervision, Validation, Writing – review & editing.

## Supplementary material

Supplementary Figure 1 Gating strategy.
