# Supplementary material for: Low levels of peripheral blood activated and senescent T cells characterize people with HIV-1-associated neurocognitive disorders
Source: Front Immunol. 2023 Oct 25;14:1267564. doi: 10.3389/fimmu.2023.1267564 (PMC10634248; doi:10.3389/fimmu.2023.1267564)
Supplement: Supplementary Figure 1 — Gating strategy. [file Image_1.pdf]

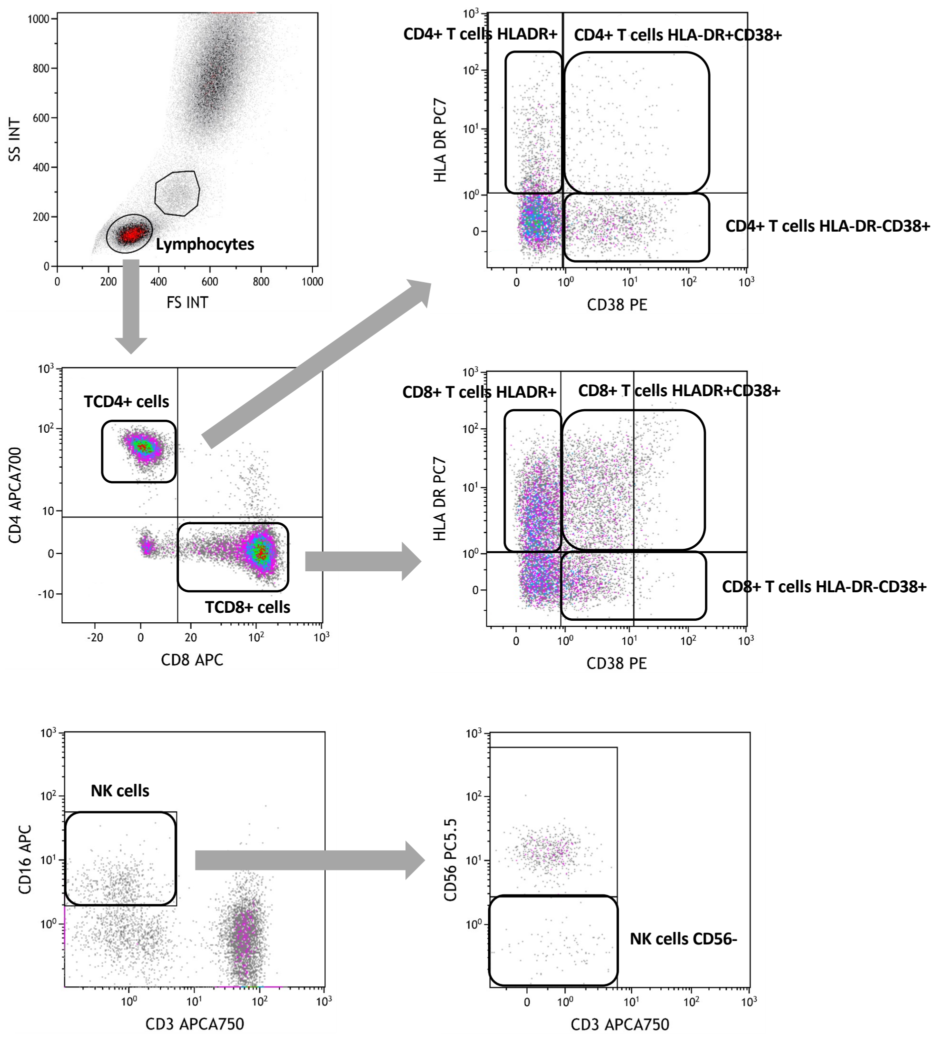

Supplementary Figure 1. Representative gating strategy for the identification of activated T4 and T8 cells, and of CD56- NK cells.
